# Passive Water Filtration Enhances the Detection of Endangered Fish Species in Rivers: *Anguilla anguilla* and *Salmo salar* Monitoring Using qPCR

**DOI:** 10.1002/ece3.73647

**Published:** 2026-08-18

**Authors:** Irene de Carlos Fernández, Alba Ardura, Sara Fernández

**Affiliations:** ^1^ Department of Functional Biology University of Oviedo Oviedo Asturias Spain; ^2^ Edificio de Investigación – 2ª Planta Universidad de Oviedo – Campus de Mieres Mieres Asturias Spain

**Keywords:** diadromous species, endangered species monitoring, environmental DNA, passive filtration methods, qPCR, rivers

## Abstract

The assessment of vulnerable species is crucial for population management. Environmental DNA (eDNA) provides multiple advantages in the monitoring of endangered populations as it is a non‐invasive and highly sensitive technique that causes minimum disturbances and allows the detection even in cases of low population numbers. *Anguilla anguilla* and *Salmo salar* are two diadromous species exposed to several threats both in the sea and rivers. They are cataloged as Critically Endangered and Near Threatened respectively (Interntional Union for Conservation of Nature) so it is especially important to use indirect sampling methodologies to monitor their populations. Here, quantitative PCR in environmental samples was performed using two different sampling techniques, one active in situ filtration system and one passive system with metaprobes deployment. Moreover, we have compared the DNA quantity and number of individuals obtained with electrofishing in the case of *Salmo salar* . We found that metaprobes are more efficient in DNA capture and suggest their incorporation in future monitoring programs aiming scarce species detection. Results also showed a trend where higher salmon abundance is associated with higher eDNA concentration when using filtered water. It is demonstrated that both methods are useful for salmon and eel monitoring, and we suggest prioritising metaprobes in cases of low abundance species detection and water filtration as a quantitative snapshot of DNA concentration.

## Graphical abstract

This study assesses the effectiveness of environmental DNA (eDNA) for monitoring the critically endangered *Anguilla anguilla* and the near‐threatened *Salmo salar* using quantitative PCR. Active in situ water filtration and passive metaprobe deployment were compared, showing that metaprobes provided greater sensitivity for low‐abundance species, while filtered water offered a quantitative association with species abundance. These findings support the integration of metaprobes into monitoring programs and highlight the complementary role of water filtration for abundance estimation in vulnerable populations.

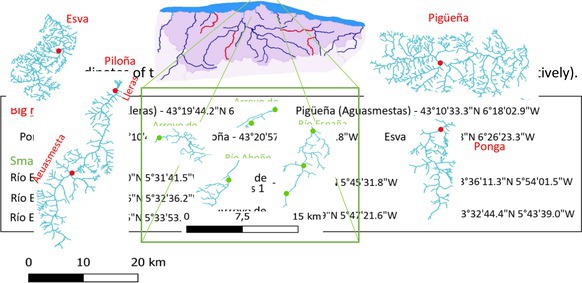

## 1 Introduction

Environmental DNA (eDNA), the genetic material that organisms release into their surroundings through processes such as excretion, shedding of cells, and decomposition (Deiner et al. 2017; Taberlet et al. 2018), could be part of the tool kit to assess vulnerable species status and the efficacy of conservation measures. The target species can be detected from the DNA present in environmental samples, without capturing or visually observing them (Valentini et al. 2016). Furthermore, eDNA techniques have proven highly sensitive at detecting low‐abundance species (Deiner et al. 2017), making them especially relevant for declining populations where traditional methods can be more limited.

The collection and analysis of eDNA are also fast and cost‐effective, they require fewer human effort and less specialized equipment (Taberlet et al. 2018). This could facilitate broader surveys across larger and even remote geographical areas, where conventional methods may be impractical or expensive. In addition, the non‐invasive nature of eDNA monitoring is beneficial from both ethical and conservation views, since it minimizes the disturbance to fragile species while still yielding information about their distribution (Valentini et al. 2016). eDNA monitoring has potential to either replace or complement traditional fish population assessment methods (Hallam et al. 2021; Jerde 2021; Spear et al. 2021).

One of the most common and straightforward methods for capturing eDNA in aquatic systems is water filtration, which involves the pumping or vacuuming of water through filters with small pore sizes (Wittwer et al. 2018). This approach has proven to be effective for assessing biodiversity in various habitats, although it often needs strict sterile procedures to avoid contamination (Taberlet et al. 2018). Recent studies have found that leaving absorbent materials in the water over extended periods of time can substantially increase eDNA recovery, since these matrices continuously accumulate genetic material passively instead of just relying on single, short‐term filtrations (Cananzi et al. 2025). One promising innovation is the “metaprobe,” a low‐cost, 3D‐printed device equipped with absorbent rolls of gauze, which have been used inside fishing nets or towed gear (Maiello, Bellodi, et al. 2024). As the net passes through large areas, the gauze continuously collects suspended DNA. This passive approach also reduces cost and effort, making it a good candidate for fish monitoring.

In recent years, eDNA has been employed for detecting the Atlantic salmon and European eel, demonstrating the efficacy of qPCR‐based methods in qualitative detection and semi‐quantitative abundance estimations (Fauchet et al. 2025; Fernandez et al. 2023; Williams et al. 2021; Atkinson et al. 2018; Itakura et al. 2019). These studies have correlated the eDNA data obtained through water filtration with fish abundance measured through electrofishing, revealing positive correlations between them. However, there is no data yet on the use of metaprobes as passive eDNA samplers for this purpose in riverine environments. As this is a newly developed methodology, there is not much information on its effectiveness.

The commonly used techniques to quantify numbers of both species include methods that require fish capture, extracting the individuals from rivers, such as electrofishing or fyke nets, and often lead to high mortalities and stress of the animal (Fernandez et al. 2023; Snyder 2003; McMichael et al. 1998). Moreover, these methodologies require a great effort in terms of time and money (Itakura et al. 2019) and they cannot be employed everywhere as the sampling sites for electrofishing need a shallow area preferably with slow flow, since high flow negatively impacts the estimation of fish abundance (Kambestad et al. 2024; Näslund et al. 2024). Furthermore, they have been reported to yield non representative results when population densities of target species are low (Penaluna et al. 2021). It is therefore important to incorporate other methodologies in order to assess both species distribution and population numbers.

Diadromous species are species that migrate between marine and freshwater environments to complete their life cycles (Verhelst et al. 2021). Populations of these migratory species have significantly declined in the last decades. A key aspect affecting them is habitat fragmentation, since these species need connectivity between and within environments to successfully migrate and complete their life cycles. Moreover, they are exposed to both sea and river threats (Lassalle et al. 2008; Ouellet et al. 2022).

Two of the most vulnerable diadromous species are the Atlantic salmon ( *Salmo salar* ) and the European eel ( *Anguilla anguilla* ). They have both experienced major population declines in recent years, which can be mainly attributed to human activities (Atkinson et al. 2018; Fernandez et al. 2023).

The Atlantic salmon is a species native to the Atlantic coast of Europe and North America (Thorstad et al. 2021). This species is anadromous, with a juvenile phase spent in rivers, an early adult phase in the ocean for feeding and finally a migration back to fresh waters for spawning (Rikardsen et al. 2021). The ecological and economical importance of Atlantic salmon makes this species as one of the most studied (Torrissen et al. 2011; Levi et al. 2019). For many years, salmon populations have been heavily impacted by intensive fisheries and aquaculture (Atkinson et al. 2018; Dempson et al. 2024). Declines have been especially severe in its southern distribution, where rising water temperatures linked to global warming have transformed many habitats making them unsuitable for salmons (Jonsson and Jonsson 2009; Nicola et al. 2018). The International Union for Conservation of Nature (IUCN) currently classifies Atlantic salmon as Near Threatened worldwide and Vulnerable in Europe. If this downward trend in population numbers continues, its conservation status will likely worsen further.

The European eel habitat range goes from Europe to North Africa (Bevacqua et al. 2015). This catadromous species spawns in the Sargasso Sea, migrates to European and North African rivers to feed and mature, and then returns to the Sargasso for reproduction (Wright et al. 2022). Like Atlantic salmon, the European eel is an important food source that has been subject to heavy fishing pressure, while river engineering and pollution have further reduced its natural habitat (Marohn et al. 2025). Barriers such as dams disrupt migration routes, and contaminants in the water can harm development. These factors, along with illegal trafficking of juvenile eels, have contributed to a severe decline in abundance (Alonso and van Uhm 2023). Consequently, the IUCN now classifies the European eel as Critically Endangered.

In the frame of concerning conservational status, efforts need to be made to stop the declining tendency of both species. The European Union created an eel management plan whereby every country within the EU had to implement measures to reduce fisheries exploitation and minimize the impact of habitat destruction to recover eel stocks (van Gemert et al. 2024; Delrez et al. 2021). In the case of the Atlantic salmon, some studies suggest that similar measures should be implemented, such as limiting dam construction, reducing fishing pressure, and introducing hatchery stocking (Hare et al. 2019).

To fully address whether these management actions are contributing to the species conservation, it is important to know the current distribution of the species and to assess population numbers.

In this study, we aim to test the effectiveness of the recently developed metaprobe sampling technique in river environments. Deploying metaprobes across various rivers, we will assess whether it is a better method for capturing eDNA than the conventional water filtration. Finally, we also seek to determine whether metaprobe and water filtration based eDNA concentrations correlate with fish abundance estimates from electrofishing, particularly focusing on Atlantic salmon. This will add information on the discussed issue of if these methods could be used for determining Atlantic salmon presence and density and provide a first exploratory assessment of the effectiveness of the recently developed metaprobe approach under real riverine conditions.

## 2 Materials and Methods

### 2.1 Locations of the Study

We conducted this study in twelve riverine systems of Asturias, located in northern Spain, focusing on sites known for their ecological importance to Atlantic salmon ( *Salmo salar* ) and European eel ( *Anguilla anguilla* ) (Lobon‐Cervia and Iglesias 2008; Cloud and Thorgaard 1993). The sampling sites were chosen from locations that are part of the annual electrofishing monitoring program coordinated by the Asturian regional government.

We selected sampling points in tributaries of two major river systems, the Nalón and Sella rivers, which differ in their degree of anthropogenic impact. The Sella River is known for having relatively good environmental conditions. Meanwhile, the Nalón River is more influenced by industrial and urban development taking place in its surroundings (Fernández et al. 2018). These rivers are known to host the highest population densities of European eel in the region, and they also support significant populations of Atlantic salmon (Lobon‐Cervia and Iglesias 2008; Cloud and Thorgaard 1993), making them suitable for comparative eDNA analysis.

**FIGURE 1:**
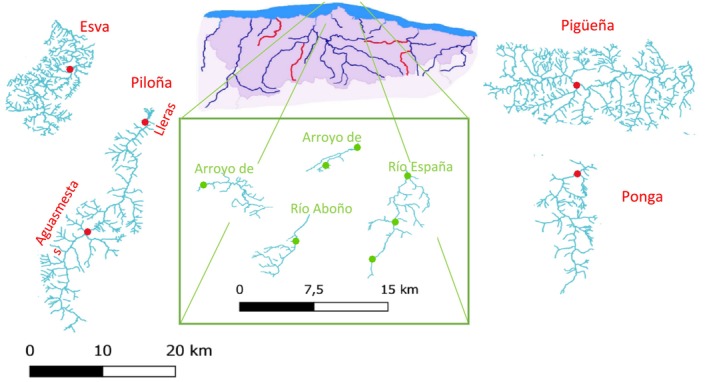
Sampling sites located in two river basins (Nalón and Sella) and several smaller coastal streams in Asturias. The figure shows the sampling points of the main tributaries (Ponga, Piloña, Pigüeña, and Esva) in red, as well as additional sampling points in coastal rivers (Arroyo de Vioño, Río Aboño, Arroyo de Reguera O de Linares, and Río España) in green.

Within these basins, the following tributaries were selected: Ponga, Piloña, Pigüeña (including sampling points in Aguas Mestas and Lleras), and Esva (Figure 1 and Figure S1). Sampling points within these tributaries included main river sections where electrofishing surveys have previously confirmed the presence of the target species.

In addition to the electrofishing monitoring sites, we also selected several smaller coastal rivers where European eels were observed actively entering the system (Rivas‐Iglesias et al. 2025). These included Arroyo de Vioño, Arroyo de Reguera O de Linares, Río España, and Río Aboño (Figure 1 and Figure S1). These smaller riverine systems are particularly relevant for eel monitoring, as previous studies suggest that eels frequently utilise low‐order streams as critical habitats, often overlooked by conventional fishery assessments (Özdilek and Özdilek 2020; Höhne et al. 2024; Griffiths et al. 2023).

### 2.2 Field Sampling

#### 2.2.1 eDNA Collection

For eDNA collection, two sampling strategies were applied across all sampling sites to compare their effectiveness in detecting target species. The variation in the number of samples among sites reflects logistical constraints inherent to field‐based sampling, as well as the opportunistic nature of the dataset used in this study. Sampling details are described below.

##### 2.2.1.1 Vacuum Filtering

River water samples were collected immediately before electrofishing took place, in order to avoid sediment disturbances. One sample was collected in small coastal rivers and three water samples per site were collected in bigger rivers where electrofishing was conducted: one in the middle of the river (M), one on the left bank (O1), and one on the right bank (O2). At each point of each site, 2 L of water were vacuum filtered using 0.22 μm pore size, 47 mm diameter filter membranes (Pall Corporation, Life Sciences, Ann Arbor, MI, USA). Filtration was performed in situ, and all equipment—including filtration apparatus, forceps, and surfaces—was cleaned between samples using a 10% bleach solution to minimize cross‐contamination. To control for contamination, filtration blanks consisting of 1.5 L of distilled water were filtered under the same conditions after each sampling event. Each filter was stored and kept in ice until frozen in the lab within the next 2 h maximum to avoid DNA degradation.

##### 2.2.1.2 Metaprobes Deployment

**Image 1:**
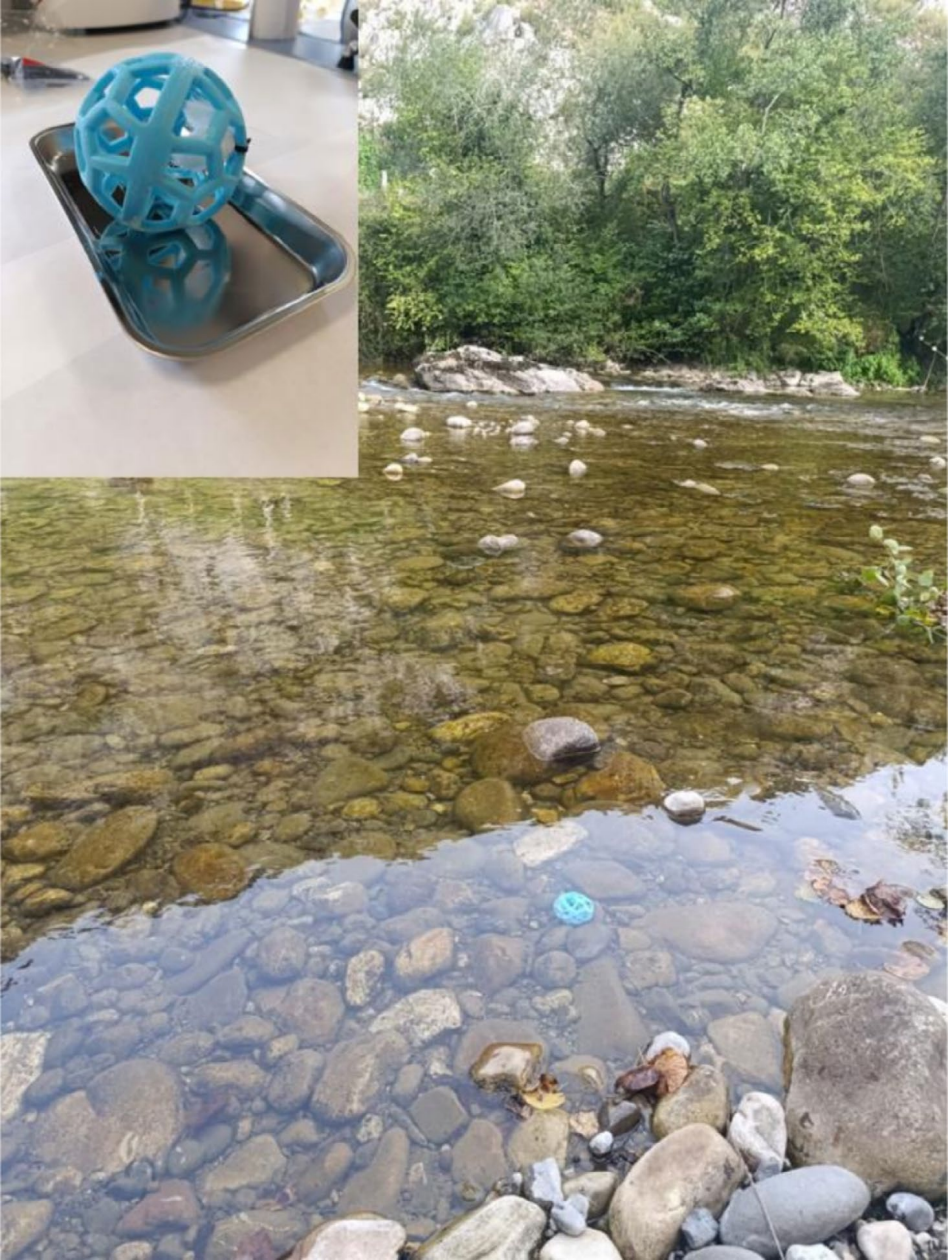
Metaprobe preparation in the lab and placed in the field.

DNA was collected passively by placing gauze‐based metaprobes (Maiello, Talarico, et al. 2024; Maiello, Bellodi, et al. 2024; Albonetti et al. 2023) in the river for 24 h (Image 1). These metaprobes consisted of 3 gauzes, each of which contained 1 g of sterile cotton wrapped in three layers of sterile gauze (10 × 10 cm mesh) and secured inside a perforated plastic sphere using zip ties, following protocols established in previous studies (Maiello, Talarico, et al. 2024; Maiello, Bellodi, et al. 2024; Albonetti et al. 2023).

In bigger rivers, where electrofishing programs were planned, three metaprobes per site were deployed, and three 2 L water samples collected. This distribution was used to assess whether eDNA concentrations differed between mid‐channel and riverbank habitats when using this collection method not previously used in river systems (Maiello, Talarico, et al. 2024; Maiello, Bellodi, et al. 2024; Albonetti et al. 2023), as well as to compare the results of the new method with water samples collection. After 24 h, they were retrieved immediately before electrofishing.

In smaller rivers, where electrofishing did not take place, one metaprobe was deployed per site as they were considerably smaller and there were also spatial constraints. Each metaprobe was attached to a rope and submerged at its designated location, ensuring constant exposure to the river's flow. The probes were collected after 24 h in the river.

The gauzes were cut in half and stored in 15 mL tubes and bead tubes in situ. All samples were transported to the lab and stored in a −20°C freezer until DNA was extracted the following day.

#### 2.2.2 Electrofishing

After eDNA collection with vacuum filtering and metaprobes retrieval, electrofishing was conducted by the regional government of Asturias region. This program has been carried out periodically at predetermined sites within a regional river monitoring network since 2011 (Fernandez et al. 2023). Electrofishing was conducted by applying the same sampling effort in all the sites. Three‐pass electrofishing covering the whole river from bank to bank was completed at each sampling point, without replacement. This approach involves sampling the same river reach in successive passes, capturing fish during each pass with a short interval between passes (e.g., 15–30 min) to allow redistribution, and is widely used in salmonid monitoring (Kambestad et al. 2024). Fishes were taken out from the river with a handle net and placed in containers with water and air supply until the end of the electrofishing passes. The individuals were counted and measured, before being released back into the same river area.

### 2.3 eDNA Extraction

eDNA of the gauzes and water filters was extracted following the DNeasy Blood and Tissue Kit (Qiagen, Hilden, Germany) protocol, with a minor modification to adjust for eDNA extraction. 800 μL of a solution consisting of 80 μL of proteinase K and 720 μL of ATL Buffer was poured onto the filter or gauze inside the bead tube. The bead tube containing the filter/gauze was shaken for 10 min to facilitate the lysis of cellular debris present on the filter. The tube was then incubated at 56°C for 3 h and then the liquid was collected after lysis. The process was continued and the final volume of eDNA was eluted in 200 μL of AE buffer, following the manufacturer's protocol. To check for cross‐contamination during the extraction, eDNA was simultaneously extracted from DNA‐free distilled water (extraction negative control) as one sample for every extraction procedure.

All pre‐PCR steps were conducted inside the laminar flow cabinet after 20 min of UV light decontamination and the post‐PCR steps were done in a separate laboratory unit. Extractions were quantified using Qubit dsDNA HS Assay Kit (Invitrogen, Carlsbad, CA, USA) (Table S1). Extractions that yielded no DNA were removed from posterior analysis.

### 2.4 Quantitative PCR (qPCR)

The eDNA samples were assayed using specific primers and a hydrolysis probe on a 7900 HT Fast Real‐Time PCR system (Life Technologies Inc., Applied Biosystems, Carlsbad, CA, USA). A fragment of the mitochondrial gene cytochrome oxidase subunit 1 (COI) was amplified and quantified using the following probe and primers for Atlantic salmon and European eel, respectively: forward primer, 5′‐CGC CCT AAG TCT CTT GAT TCG A‐3′; reverse primer, 5′‐CGT TAT AAA TTT GGT CAT CTC CCA GA‐3′; probe, 5′‐5ATTO550N AGA ACT CAG CCA GCC TG‐3′ (Atkinson et al. 2018); forward primer, 5′‐GGA GCT GGT ACA GGC TGA ACT G‐3′; reverse primer, 5′‐AGT GAG AAA ATT GTC AGG TCA ACA GA‐3′; probe, 5′‐6‐FAMTGG CTG GAA ACT TAG CCC ACG CC BHQ1‐3′ (Fernandez et al. 2023). Plates were prepared inside a laminar flow cabinet previously decontaminated with UV light for 20 min. Each 15 μL qPCR reaction contained 6 μL of extracted eDNA solution, 5.92 μL of TaqMan Environmental Master Mix 2.0 (Life Technologies, Carlsbad, CA, USA), 0.474 μL of each primer (10 mM), and 0.474 μL hydrolysis probe (10 mM). qPCR was performed in triplicate for each eDNA sample under the following conditions: 2 min at 50°C, 10 min at 95°C, and 40 cycles of 15 s at 95°C and 1 min at 60°C. Sterile distilled water (2 μL) was also analyzed in all rounds of qPCR as a negative control.

### 2.5 Data Analysis

All statistical analyses were conducted using R software (v4.3.3). DNA quantity was calculated using a standard curve included in each qPCR plate. These curves were generated from serial dilutions of a positive control of either *S. salar* or *A. anguilla* , and Cq values were regressed against the log_10_ of the known copy number (*Ct* = *β*_0_ + *β*_1_·log_10_ [copies per reaction]). The resulting regression model was used by the qPCR software to interpolate the number of copies in each sample. Amplification efficiency was calculated from the slopes using the equation: $E=\left(10^{-1/\text{slope}}-1\right)*100$. And the coefficient of determination (*R* ^2^) was used as an indicator of linearity.

Given the novelty of the metaprobe sampling method, linear mixed‐effects models were employed to evaluate differences in eDNA capture between different probe positions, as well as to compare the performance of metaprobes with traditional water filtration. To assess whether the position of the metaprobes (middle vs. riverbanks) influenced the quantity of captured eDNA, a linear mixed‐effects model was fitted with the following structure: DNA quantity∼Group + species + (1∣Location) where the DNA quantity represents the number of copies obtained, Group represented a categorical variable indicating the metaprobe position: O1 (left bank), M (middle), or O2 (right bank), species was a categorical variable that accounted for the differences in the DNA amplified depending on the species (Atlantic salmon or European eel) and Location was included as a random intercept to account for variation across different sampling sites.

Individual metaprobes consisted of three gauze layers, each processed independently. For each gauze, three qPCR replicates were performed, and the mean quantity values of these replicates were used as the input for the model. Thus, for each sampling site, we obtained three DNA quantities per metaprobe position (one per gauze layer), resulting in nine values per site.

To compare the eDNA capture efficiency of metaprobes relative to conventional water filtration, a second linear mixed‐effects model was fitted with the following structure: DNA quantity∼Method + Species + River + (1∣Location), where DNA quantity represented the quantity values obtained from qPCR, Method was a categorical variable with two levels: “Metaprobe” (gauze‐based sampling) and “Water” (filtered water samples), species was a categorical variable that accounted for the differences in DNA quantity depending on the species (Atlantic salmon or European eel), “River” was a categorical variable that accounts for whether the sampling was done in big or small rivers and Location was again treated as a random intercept to account for site‐specific variability. This analysis was performed using data from both large and small rivers. However, as only one metaprobe was deployed in small rivers, we restricted the comparison to the middle‐position metaprobes in larger rivers. We averaged the DNA quantity of the three gauzes to obtain a single value per site. Similarly, water sample replicates were averaged to obtain a single value per site for comparative purposes. Statistical significance was evaluated at a *p*‐value threshold of 0.05.

Additionally, for rivers where electrofishing was employed, we calculated the correlation between DNA quantity values and the number of individuals of *S. salar* . For each location, we correlated these values of the gauzes in the middle metaprobes having higher DNA concentration with the total number of Atlantic salmon recorded during electrofishing. The same procedure was applied to the filtered water samples, where we correlated Ct values from the water replicates with the electrofishing counts for the species. Pearson's correlation coefficients (*r*) were calculated along with their corresponding *p*‐values to evaluate the strength and significance of these relationships.

## 3 Results

Standard curves generated for each qPCR plate showed high linearity (*R* ^2^ = 0.98–0.99). Amplification efficiencies were generally within the expected range (96%–104%).

### 3.1 Metaprobes Location

**FIGURE 2:**
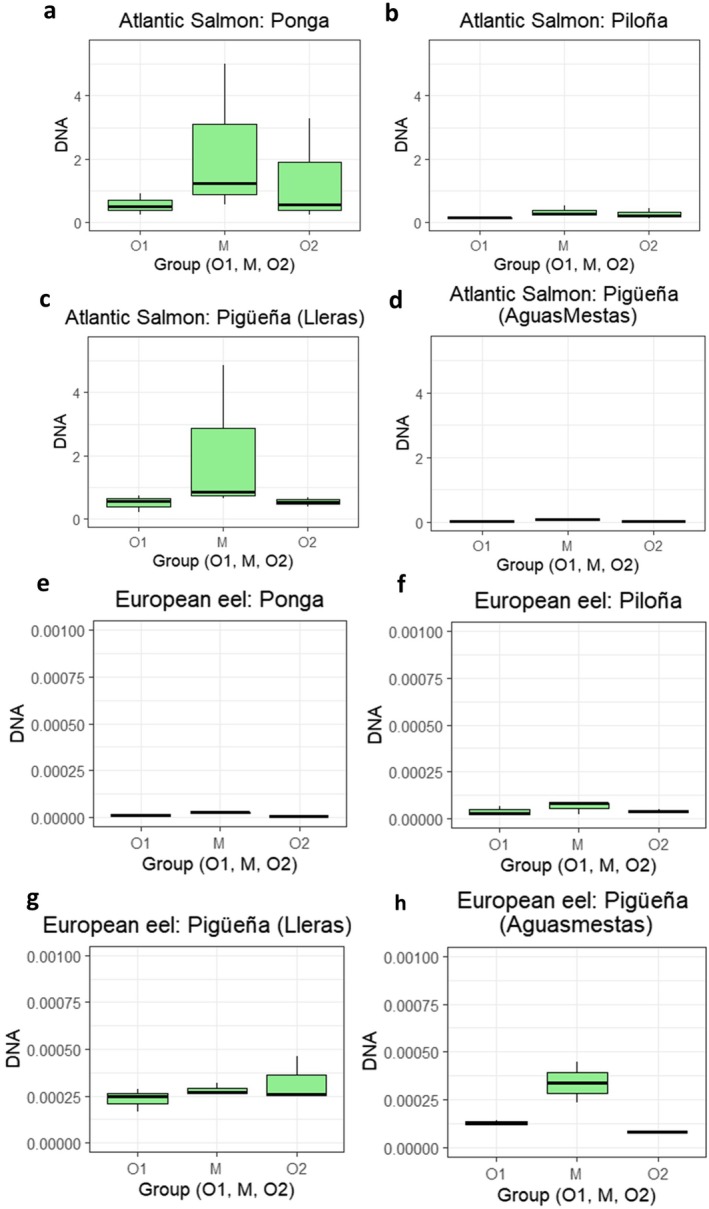
Boxplots of DNA quantity for salmon and eel, collected at three sampling positions—left bank (O1), middle (M), and right bank (O2)—in four tributaries. Panels: (a) Atlantic salmon–Ponga, (b) Atlantic salmon–Piloña, (c) Atlantic salmon–Pigüeña (Lleras), (d) Atlantic salmon–Pigüeña (AguasMestas), (e) European eel–Ponga, (f) European eel–Piloña, (g) European eel–Pigüeña (Lleras), and (h) European eel–Pigüeña (AguasMestas). The horizontal line within each box represents the median DNA quantity, the box spans the interquartile range (IQR), and the whiskers extend to the furthest points that are not considered.

The comparison of eDNA capture efficiency across different metaprobe positions (middle of the river vs. riverbanks) did not show differences in DNA quantity amplified using qPCR (Figure 2). The mean values obtained from metaprobes placed in the middle of the river (M) were higher compared to those from the left (O1) and right riverbanks (O2), meaning that there is a higher concentration of Atlantic salmon or European eel eDNA in those points. However, the linear mixed‐effects model (DNA ~ Group + Species + (1|Location)) was not significant, showing no significant effect of the metaprobe position (Table 1).

**TABLE 1:** Results of the linear mixed‐effects model (LMM).

LMM	Effect	Estimate	Std. error	df	*t* value	*p*
	(Intercept)	−9.081262	1.522668	3.646795	−5.964	0.0053
	Group M	0.832007	0.727417	61.931806	1.144	0.2571
	Group O_2_	0.292859	0.754652	61.950848	0.388	0.6993
	Species	0.073249	0.006322	63.837255	11.587	< 2e‐16

*Note:* log_10_ (DNA concentration)~Group + Species + (1|Location). We report the effect, the estimate, the standard error (SE), degrees of freedom (df), *t*‐value and *p*‐value and for the post hoc Tukey test the contrast.

### 3.2 eDNA Collection Methods

**FIGURE 3:**
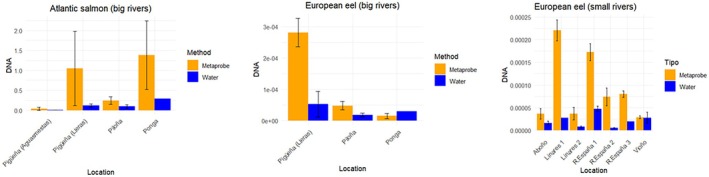
Bar charts comparing mean values for DNA quantity using qPCR (± standard error) from two methods, metaprobes and filtered water, across various sampling sites in the two considered species ( *Salmo salar* and *Anguilla anguilla* ).

For the majority of locations, the DNA found from metaprobes was higher than those from filtered water (Figure 3). As explained in methods, we also added the data collected from small rivers for European eels, as both metaprobe and water filtration methods were employed here. The linear mixed‐effects model (DNA~Method + Species + River + (1|Location)) indicated a significant effect of the sampling method (*p* = 9.51e‐05), with metaprobes showing significantly higher values compared to filtered water samples, suggesting higher eDNA capture (Table 2). The species effect was also statistically significant (*p* = 0.2.36e‐14) but not the river size (*p* = 0.957), indicating that the differences in DNA quantity were consistent across river sizes but not for the species (Table 2). Interaction terms were initially included in the model but were not statistically significant and led to overparameterisation. Therefore, a reduced model including only main effects was selected.

**TABLE 2:** Results of the linear mixed‐effects model (LMM) testing the effects of sampling type, species and river size on log‐transformed environmental DNA concentration.

LMM	Effect	Estimate	SE	df	*t* value	*p*
	(Intercept)	−4.7747	0.2110	17.3949	−22.632	2.39e‐14
	Method (Metaprobe)	0.6632	0.1339	18.5032	4.953	9.51e‐05
	Species (Salmon)	3.5682	0.1854	19.9462	19.250	2.36e‐14
	River size (small)	−0.0139	0.2541	13.6929	−0.055	0.957

*Note:* log_10_ (DNA concentration)~Method + Species + River + (1|Location). DNA concentrations were log_10_‐transformed prior to analysis. Metaprobe samples yielded significantly higher DNA concentrations than water samples (estimate = 0.66 ± 0.13 SE, *p* < 0.001), corresponding to approximately a 4.6‐fold increase in detected DNA. Atlantic salmon samples showed substantially higher DNA concentrations than European eel (*p* < 0.001), while river size had no significant effect.

### 3.3 Fish Abundance and eDNA Quantity

The results of the Pearson correlation analysis indicated a positive and significant correlation between eDNA quantity and the number of salmon individuals detected, suggesting that higher abundance is associated with higher eDNA concentrations (*r* = 0.6216, *p*‐value = 0.0309, *t* = 2.5099, df = 10).

## 4 Discussion

### 4.1 Metaprobes Location

The novel metaprobe approach tested here, originally developed for marine ecosystems (Maiello, Talarico, et al. 2024; Maiello, Bellodi, et al. 2024; Albonetti et al. 2023), showed a potential for its use in riverine environments. In contrast to previous commonly used water filtration methods, which often reported higher fish eDNA concentrations along riverbanks (Jane et al. 2015), our results suggest that placing metaprobes in the middle of the river could yield higher eDNA capture. A possible explanation is that metaprobes remain in situ for 24 h, effectively acting as an obstacle through which fast currents and a larger volume of flowing water, and thus more genetic material is retained. By comparison, a single instantaneous water grab at the riverbanks may capture eDNA that is briefly retained by slower currents or some obstacles. But in places where the currents are fast or with high flow, this approach could have less efficient eDNA captures as the eDNA is more diluted (Curtis et al. 2021; Jane et al. 2015).

The observed trend favouring the mid‐channel location highlights the advantage of deploying passive sampling devices like the metaprobe for long periods of time in fast water flowing sections of rivers. Although some studies are currently being developed (Cananzi et al. 2025), future work should expand the range of sites and species tested using qPCR to further confirm this trend.

### 4.2 eDNA Collection Methods

Metaprobes consistently yielded higher DNA quantities, indicating higher capture efficiency than traditional water filtration, in both Atlantic salmon and European eel. The ability of metaprobes to detect eDNA across distinct species suggests that this approach could be used for other species not evaluated here.

A study by Chen et al. (2022) showed that 4 h of deployment of absorbent materials yielded an amount of eDNA comparable to filtering 1 L of water. They also observed that even after 72 h, those absorbent materials had not reached eDNA collection saturation. Another study by von Ammon et al. (2025) also found that deploying various absorbent materials for several hours resulted in greater eDNA yields compared to water filtration methods. Taken together, these findings suggest that the metaprobe deployment time was a key factor in its higher eDNA capture compared to active water filtration.

Apart from yielding more eDNA, the metaprobe offers other practical advantages over water filtration. Traditional filtration methods require costly equipment and the transportation of water samples to the lab or pumping systems into the field (Bessey et al. 2021). These approaches also generate significant plastic waste and can be difficult to implement in turbid waters or rivers with debris. Moreover, depending on the river characteristics 1 L of water might not be enough to capture enough material to be representative of the diversity (Bessey et al. 2021). However, water filtration allows for the processing of a known volume of water that is taken as an instantaneous water grab, which gives more control and precision when trying to estimate the biomass present at that site. In this aspect, standardization with metaprobes can be more difficult.

In contrast, the metaprobe is more cost‐effective and easier to deploy and retrieve. Water conditions do not pose a problem as there is no filtration of water involved, and this method minimizes both the amount of waste produced and the equipment required for sample processing. This approach enables visiting multiple sites in a single day, minimizing the material prices and the time spent in each sampling site, however, two visits to sampling point are necessary. Furthermore, if the deployment of absorbent materials can capture similar yields than filtered water in 4 h, they could be easily used along with electrofishing. To fully develop this methodology, future studies are necessary to determine the optimal or sufficient deployment time for metaprobes under varying field conditions. Considering different physico‐chemical parameters and its effect over the longevity of eDNA in the surrounding environment, because the water quality can alter eDNA persistence, and that eDNA is influenced by various abiotic and biotic factors, such as temperature, pH or bacterial composition among others (McCartin et al. 2022; Joseph et al. 2022).

Previous studies have shown that eDNA sampling can be dependent on the nature of the biodiversity to describe. Cananzi et al. (2025) have found that both methods detected a similar number of vertebrates to filters, but with lower performance in the detection of fish for passive methods. Finally, continuous eDNA collection by passive methods has shown to be more effective than instantaneous water sampling at capturing low abundance and ephemeral species in natural waters, facilitating more efficient and convenient eDNA‐based biodiversity surveillance and rare species detection (Chen et al. 2024).

Therefore, it seems essential to develop further studies to evaluate the performance of passive systems considering different aquatic environmental conditions, sampling duration, as well as different species with different behaviours and densities, also combining different primer sets, in order to optimize their application according to the environmental conditions and the research objective.

### 4.3 Fish Abundance and eDNA Quantity

eDNA capturing tools could be useful to analyze the biomass present at certain environments. Our results highlighted a general expected trend where higher fish abundance is associated with higher eDNA concentration consistent with the notion that greater biomass or density leads to increased DNA shedding (Barnes and Turner 2016). Filtered water samples exhibited a statistically significant positive correlation with Atlantic salmon abundance. However, the metaprobes did not reach statistical significance. This could be because, as mentioned above, metaprobes are deployed in the water for a long period of time and the water volume sampled by them is unknown. This lack of control introduces uncertainty when trying to relate eDNA concentration to organism abundance or biomass since changes in environmental conditions and stochastic species activity could affect the quantity of eDNA captured (Chen et al. 2024). Another aspect that could have affected this result is that, at the site in Esva river, metaprobes were not deployed due to time constraints, thus having less data for the correlation.

Similar studies have investigated the relationship between eDNA concentrations and biomass in other species, finding significant correlations between eDNA copy numbers and the number of individuals captured by electrofishing surveys (Itakura et al. 2019; Jacobsen et al. 2023). These findings along with our results support the potential for eDNA methods to provide biomass estimates. However, despite these significant correlations, there is considerable variability between the number of fish found and the eDNA measurements. This variability could stem from differences in river characteristics among sampling points. For instance, variations in water flow rate, temperature, pH, or turbidity among sampling points can affect how quickly eDNA degrades or disperses (Goldberg et al. 2015; Barnes and Turner 2016). It could also be due to variations in eDNA production rates among Atlantic salmons (Wilcox et al. 2016), or the possibility that electrofishing surveys fail to capture all individuals in certain rivers (Penaluna et al. 2021). Moreover, in low concentrations, the reliability of the qPCR diminishes, affecting replicability of samples with low eDNA copies which can lead to the observed variability (Guri et al. 2024). Therefore, we argue that eDNA can offer information on whether there is high or low abundance of fish, though providing an exact biomass estimate remains challenging. Additional studies are needed to present it as an alternative to electrofishing in areas where species abundance is low as it is the case of endangered species populations, as well as in rivers with difficult access or areas where conventional electrofishing surveys are not feasible.

### 4.4 Implication for Management and Conservation

As demonstrated here, metaprobes and water filtration serve as valuable tools for detecting species presence and understanding their distribution, and both could be particularly beneficial for monitoring European eel and Atlantic salmon in riverine environments. While metaprobes offer a practical, low‐effort method for presence/absence detection over extended periods, water filtration provides a more quantitative snapshot of eDNA concentration at a specific time and location and therefore is a useful tool for determining whether European eel and Atlantic salmon abundance is high or low. All this information could be beneficial for identifying critical habitats, assessing spawning success, or prioritising areas for restoration to recover Atlantic salmon and European eel stocks. It is also worthy to mention that European eel eDNA was detected even in the highest sections of the smaller coastal rivers that were sampled in this study, suggesting that these individuals use these habitats for growth and development. Several studies have reported eels migrating into upper sections of river systems, and some studies also highlight the potential of small coastal rivers in eel conservation (Copp et al. 2021; Kettle et al. 2010; Jacoby and Gollock 2014). Because European eels are classified as Critically Endangered by the IUCN, conservation measures should also extend to these smaller rivers to safeguard their populations (González‐Pola et al. 2019). For example, restricting fishing activities in these rivers, preserving or restoring connectivity and preventing the construction of infrastructures that block or alter natural flow are all important to ensure that European eels can continue to access these habitats.

## Author Contributions

**Irene de Carlos Fernández:** writing – original draft (equal). **Alba Ardura:** writing – original draft (equal). **Sara Fernández:** writing – original draft (equal).

## Funding

This work was supported by Gobierno del Principado de Asturias, GRU‐GIC‐24‐051. Ministerio de Ciencia e Innovación, PID2022‐138523OB‐I00.

## Conflicts of Interest

The authors declare no conflicts of interest.

## Supporting information


**Figure S1:** Coordinates of the sampled sites of big and small rivers (red and green respectively).

**Table S1:** Tables of all the data collected for each sampled point for Atlantic salmon and European eel in big rivers and European eel in small river.

## Data Availability

All data can be accessed in the Supporting Information—S1 of this manuscript.
