# Supplementary material for: Passive Water Filtration Enhances the Detection of Endangered Fish Species in Rivers: Anguilla anguilla and Salmo salar Monitoring Using qPCR
Source: Ecol Evol. 2026 Aug 18;16(8):e73647. doi: 10.1002/ece3.73647 (PMC13487382; doi:10.1002/ece3.73647)
Supplement: Supplementary file 1 [file ECE3-16-e73647-s001.docx]

**Appendix**

**Fig. S1**: Coordinates of the sampled sites of big and small rivers (red and green respectively).

**Table S1**: tables of all the data collected for each sampled point for Atlantic salmon and European eel in big rivers and European eel in small river.
